# A first glance on the molecular mechanisms of pheromone-plant odor interactions in moth antennae

**DOI:** 10.3389/fncel.2012.00046

**Published:** 2012-10-25

**Authors:** Sylvia Anton, Michel Renou

**Affiliations:** ^1^Faculté des Sciences, Laboratoire Récepteurs et Canaux Ioniques Membranaires, UPRES-EA 2647 USC INRA 1330, Université d'AngersAngers, France; ^2^Physiologie de l'Insecte: Signalisation et Communication, Centre de Recherches de Versailles, UMR 1272 INRA/Université Paris 6Versailles, France

**A commentary on**

**Plant odorants interfere with detection of sex pheromone signals by male *Heliothis virescens***

by Pregitzer, P., Schubert, M., Breer, H., Hansson, B. S., Sachse, S., and Krieger, J. (2012). Front. Cell. Neurosci. 6:42. doi: 10.3389/fncel.2012.00042

It is widely accepted that odorants emanating from different organic sources are interacting to elicit behaviors in animals, including insects. However, the mechanisms of such interactions are largely unknown. In insects, the most prominent examples for odor interactions are mixtures of host odors and anthropogenic repellents in blood-sucking insects such as mosquitoes (Syed and Leal, 2008) and synergistic or inhibitory interactions of sex pheromones and host or non-host plant odors in moths (Byers et al., 2004; Yang et al., 2004; Schmidt-Büsser et al., 2009; Allmann and Baldwin, 2010; Varela et al., 2011). Detection of pheromone and plant odors in moths, for instance, is known to happen via highly separated channels whose input is transmitted via labeled lines to primary and even secondary processing centers (Christensen and Hildebrand, 2002). Behavioral effects issuing from this particular example of mixture interactions have therefore been thought to occur mainly through integration in higher centers within the brain (Lei and Vickers, 2008).

The literature shows, however, that olfactory signals supposed to serve as cues for different behaviors, like sex pheromone and plant odor, interact already in the peripheral detection system (Den Otter et al., 1978; Van der Pers et al., 1980; Ochieng et al., 2002; Party et al., 2009; Hillier and Vickers, 2011; Rouyar et al., 2011; Deisig et al., 2012). Moreover the information on odor mixtures might subsequently be modified throughout the olfactory pathway (Namiki et al., 2008; Barrozo et al., 2010; Chaffiol et al., 2012; Deisig et al., 2012). The pheromone-plant odor interactions have been mainly analyzed with *in vivo* optical imaging or extra- and intracellular electrophysiological recording techniques, revealing suppressive or synergistic interactions at the cellular level. However, nothing was known so far on the molecular mechanisms involved in the observed interactions. The major hypotheses were that plant odors might interfere with pheromone binding to binding proteins or olfactory receptors in a competitive or non-competitive way. A contribution of ion channels or odorant degrading enzymes, which influence the dynamics of odor responses in olfactory receptor neurons was also considered (Pophof and Van der Goes van Naters, 2002; Ishida and Leal, 2008).

In the article published in Frontiers of Cellular Neuroscience volume 6, P. Pregitzer and co-authors confirm the inhibition of sex pheromone responses by certain plant odorants, using *in vivo* calcium imaging of the antennal lobe, i.e., responses of receptor neurons from the entire antenna in their model, the noctuid moth, *Heliothis virescens*. *H. virescens* is a favorable model to investigate molecular mechanisms underlying pheromone-plant odor interactions in antennal sensilla, because the pheromone binding protein (HvirPBP2) and the olfactory receptor (HR13) binding the major pheromone compound, *Z*-11-hexadecenal (Z11-16:Ald), have been identified (Grosse-Wilde et al., 2007). The authors profited from this knowledge to investigate effects of plant odorants alone or in combination with Z11-16:Ald on HvirPBP2 and on HR13. The tested plant odorants did not themselves bind to HvirPBP2 and did not alter binding of the main pheromone component to HvirPBP2. However, pheromone-induced responses of human embryonic kidney (HEK) cells expressing HR13 changed in a dose-dependent manner, when certain plant odorants are added. Interestingly, the same plant odorants eliciting inhibition of pheromone responses in the antennal lobe also reduced pheromone responses in the HR13-expressing cells. On the other hand, a fruit odorant, without evident behavioral significance for the moth, did neither have an effect on receptor neuron responses to the sex pheromone, nor did it change pheromone responses in HR13-expressing cells. These results are a first important step towards identifying the molecular actors involved in pheromone-general odorant interactions within the highly specific pheromone detection system on the antennae of an insect. The transport of pheromone molecules through the sensillum lymph seems not to be affected by plant odorants, but pheromone binding to membrane receptors changes in the presence of plant odorants. Although the odorant types are rather different, these effects are similar to the action of the insect repellent DEET on olfactory receptors in different mosquito species and *Drosophila melanogaster* (Ditzen et al., 2008; Bohbot et al., 2011; Bohbot and Dickens, 2012).

The study by Pregitzer et al. shows that we just begin to understand peripheral interactions of different odorants. In the future it will be exciting to see if the situation found in a heterologous system corresponds to a “real life” situation with the complex environment of an antennal sensillum, in which different molecular actors are present and where potential feedback from the antennal lobe might affect receptor neuron responses. The current results help to refine the future approaches by excluding already some players and proposing candidate molecular actors involved in environmental modulation of olfaction.
